# Clinical assessment of a non-invasive wearable MEMS pressure sensor array for monitoring of arterial pulse waveform, heart rate and detection of atrial fibrillation

**DOI:** 10.1038/s41746-019-0117-x

**Published:** 2019-05-14

**Authors:** Matti Kaisti, Tuukka Panula, Joni Leppänen, Risto Punkkinen, Mojtaba Jafari Tadi, Tuija Vasankari, Samuli Jaakkola, Tuomas Kiviniemi, Juhani Airaksinen, Pekka Kostiainen, Ulf Meriheinä, Tero Koivisto, Mikko Pänkäälä

**Affiliations:** 1 Department of Future Technologies, University of Turku, 20500 Turku, Finland; 2 Department of Bioengineering, Imperial College London, London, SW7 2AZ UK; 3 Murata Electronics Oy, 01621 Vantaa, Finland; 4 Heart Center, Turku University Hospital and University of Turku, 20521 Turku, Finland; 5 Harvard Medical School, MacRae Laboratory Brigham and Women’s Hospital, Boston, MA 02115 USA

**Keywords:** Diagnosis, Biomedical engineering

## Abstract

There is an unmet clinical need for a low cost and easy to use wearable devices for continuous cardiovascular health monitoring. A flexible and wearable wristband, based on microelectromechanical sensor (MEMS) elements array was developed to support this need. The performance of the device in cardiovascular monitoring was investigated by (i) comparing the arterial pressure waveform recordings to the gold standard, invasive catheter recording (*n* = 18), (ii) analyzing the ability to detect irregularities of the rhythm (*n* = 7), and (iii) measuring the heartrate monitoring accuracy (*n* = 31). Arterial waveforms carry important physiological information and the comparison study revealed that the recordings made with the wearable device and with the gold standard device resulted in almost identical (*r* = 0.9–0.99) pulse waveforms. The device can measure the heart rhythm and possible irregularities in it. A clustering analysis demonstrates a perfect classification accuracy between atrial fibrillation (AF) and sinus rhythm. The heartrate monitoring study showed near perfect beat-to-beat accuracy (sensitivity = 99.1%, precision = 100%) on healthy subjects. In contrast, beat-to-beat detection from coronary artery disease patients was challenging, but the averaged heartrate was extracted successfully (95% CI: −1.2 to 1.1 bpm). In conclusion, the results indicate that the device could be useful in remote monitoring of cardiovascular diseases and personalized medicine.

## Introduction

Flexible wearable devices have taken a key role in remote monitoring of vital signs while cardiovascular health monitoring is expected to witness substantial growth in the coming years.^1^ Increasing cost of treatments, aging population, and the demand for independent living are driving the growth of remote monitoring device markets.^2^ The maturing technologies now allow the integration of flexible wearable sensors to low-cost computational platforms with unprecedented capabilities to store, share, and analyze data.^3^ Wearable monitoring systems with low power consumption, ruggedness and high sensitivity are required for new continuous and unobtrusive remote health monitoring opportunities.^4^

Pressure sensors are one of the promising sensing modalities for remote health monitoring and several approaches that have been investigated recently include field-effect transistors,^5–7^ resistivity-based sensing,^8–10^ piezoelectric sensors,^11^ passive sensors with wireless read-out electronics,^12^ electronic skin applications,^13–15^ and nature-inspired sensor structures.^16,17^ These studies present great advancements in the field and measurements have been conducted for measuring the arterial or carotid pulse pressure waveforms,^5–8,11–13^ as well as intracranial pressure.^12^

Although significant progress have been made on the device and sensor development, the previous studies have not investigated capabilities beyond proof-of-concept measurements outside of laboratory. The goal of this study is to design a highly sensitive wearable device for continuous cardiovascular monitoring, and examine the device’s performance in a clinic with real patients. We demonstrate a simple process for creating a low-cost and low-power wearable tactile pressure sensor based on a microelectromechanical sensor (MEMS) elements. The applicability of the sensor is studied by carrying out arterial waveform analysis, atrial fibrillation detection, and heart rate monitoring. The arterial waveform analysis and pulse profile carries valuable information related to cardiovascular disorders, such as hypertension, arteriosclerosis, and cardiomyopathy^18–21^ and by analyzing the entire arterial waveform continuously a more complete health status can be obtained.^22–24^ On the other hand, atrial fibrillation is manifested by irregular beat intervals rather than the shape of the waveform.^25^

Currently there are several commercial devices that can capture the arterial waveform with calibrated amplitude, effectively measuring the beat-to-beat blood pressure.^26,27^ These devices are intended for clinical use in hospital environment and are expensive. Similarly, intra-arterial measurements can capture arterial waveform with correct pressure values, but this method is strictly limited to clinical environment and further hold risk for infection and discomfort.^28^

In this study, we show that it is also possible to monitor heart rate, detect arrhythmias, and extract high-quality arterial waveforms for further medical analysis with a single sensing modality wearable low-cost device with a minimal power consumption that is suitable for remote and home-monitoring applications.

## Results

### Device construction and sensor operation

**Fig. 1:**
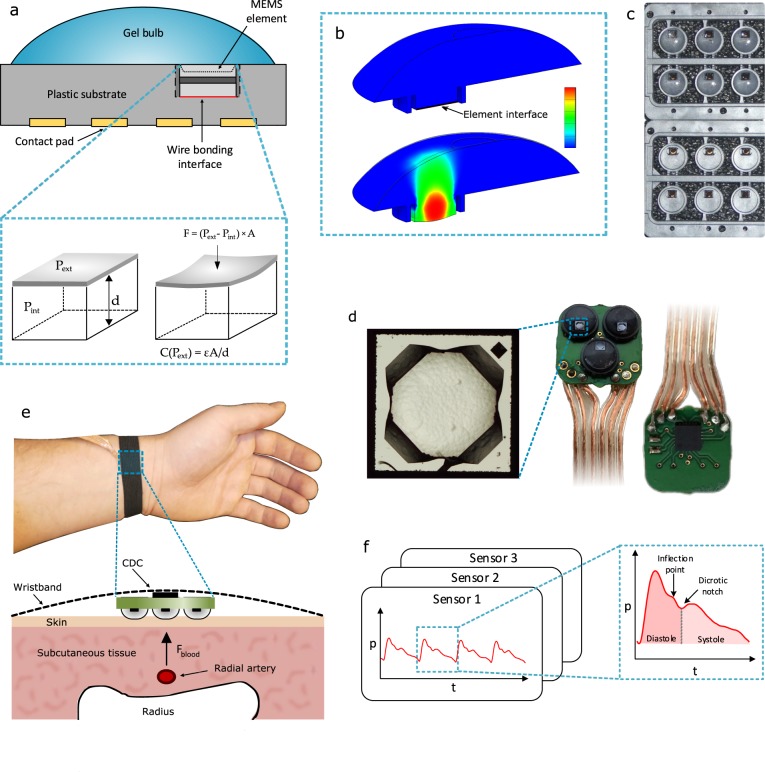
Sensory system overview. **a** Sensor operation principle where the capacitance of the element changes as a function of outer pressure deforming the diaphragm. **b** Stress analysis results; top: a cross-section of the silicone gel bulb applied on top of the sensor element and substrate, bottom: simulated displacement of the gel when uniform pressure of 100 mmHg is applied to the surface. **c** Pristine and gel-modified elements. **d** Top view microscope photograph of the square-shaped element with a side length of 1.2 mm and photographs of the assembled sensor array; top view showing the elements and backside view of the PCB showing the capacitance to digital converter. **e** The array assembled on a flexible wristband and strapped on a healthy study subject and a cross-section of the tissue at the point of measurement. Most of the force created by blood pressure in the radial artery is projected to the sensor array. **f** Illustration of the obtained signals from the array and details of the pulse profile during one cardiac cycle

A MEMS pressure sensor element (SCB10H) capable of measuring absolute pressures in the range of 0–120 kPa was selected as a starting point for the wearable sensor system.^29^ The sensor is a silicon chip with 27 µm-thick diaphragm that separates the outer and inner pressures. This diaphragm (area A) bends when an outer pressure induces a force $F = \left( {P_{{\mathop{\rm{int}}} } - P_{{\mathrm {ext}}}} \right) \times A$ on it, resulting in a change in the distance (*d*) between capacitor plates. Thus the capacitance is a function of outer pressure according to the well-known capacitive relation $C = \varepsilon A{\mathrm{/}}d$, where *ϵ* is permittivity. Figure 1 illustrates an overview of the MEMS pressure sensor setup of this study where (a) depicts the operating principle of pressure sensing element, (b) displacement simulation of the gel bulb, (c) shows a set of pristine and modified elements, (d) microscopic photograph of an element and the sensor array configuration assembled on PCB, (e) an example of the wearable device in its intended use and the coupling mechanism of the arterial pulse wave to the sensor array, and (f) recorded sensory signals and a typical pulse profile obtained from one cardiac cycle.

The MEMS elements in the array, originally intended for atmospheric measurements, were modified for wearable cardiovascular monitoring by curing a silicone gel bulb over the element. First, the sensor was fixed on 5 mm diameter substrate and gold wires were bonded from the element to the substrate. A gel bulb was manually applied over the element. The gel was then cured at 150 °C in an oven for a few minutes. Three elements were assembled as an array on a circuit board. The array configuration relaxes the requirement for accurate positioning of the device and therefore improves the reproducibility of the measurement. The capacitive-to-digital converter was soldered on the backside of the circuit board, as close as possible to the signal source to minimize the noise coupling. Behavior of the gel under uniform pressure of 100 mmHg was simulated using Autodesk Inventor 2018 stress analysis. The exerted force creates a displacement in the gel towards the elements diaphragm causing it to bend and simultaneously change its capacitance. By attaching the elements on the wrist using the wristband so that the artery is being pushed between the sensor and the wrist bone, a pulse wave can be measured.^18,19^ This is due to the pressure wave traveling across the arteries during each cardiac cycle. When this pressure wave arrives at the location of the sensor, the dilating artery creates a pressure signal which changes the capacitance of the sensor and the arterial pulse waves can be recorded continuously.

It was found that the signal quality is affected by the tightness of the strap. If the strap is attached loosely, the device produces a signal with minuscule pulse amplitudes. This is most likely due to the poor coupling of the arterial pressure signal to the sensing element. In contrast, with a very tight attachment, the artery was almost entirely blocked and the pulse waveform was by most part lost. The optimal strap tightness for monitoring the waveform was found empirically, and roughly equal strap tension was applied in each measurement without any subject-specific optimization. The strap did not cause significant discomfort for the user. However, the recording times were no longer than 10 min and for applications requiring clearly longer measurement times the user comfort should be improved using, e.g. wristwatch type designs that would be at least partly rigid and only press the sensors down where needed. Furthermore, all the used materials are skin safe and thus do not cause any skin irritation.

Another factor having a significant impact on the detected signal quality was the placement of the sensor element. The developed array configuration, however, overcomes this difficulty as it significantly simplifies the attachment procedure since proper signal is required only from one element. Moreover, having several gel-covered elements resulted in a good placement stability, i.e. sensor array remained in its placed position regardless of hand movement or tension, but such movement and tension do cause a significant changes in the signal amplitude. This is not surprising as forces created by the movement of the radial artery are minuscule. In practice, high-quality recordings can be made when subjects are staying idle and by automatically removing motion artifacts when they are not.

The use of this new single modality sensor, that has been clinically tested for continuous monitoring of arterial pulse profile, atrial fibrillation, and heart rate, holds promise for future personalized medicine applications.

### Device characterization

**Fig. 2:**
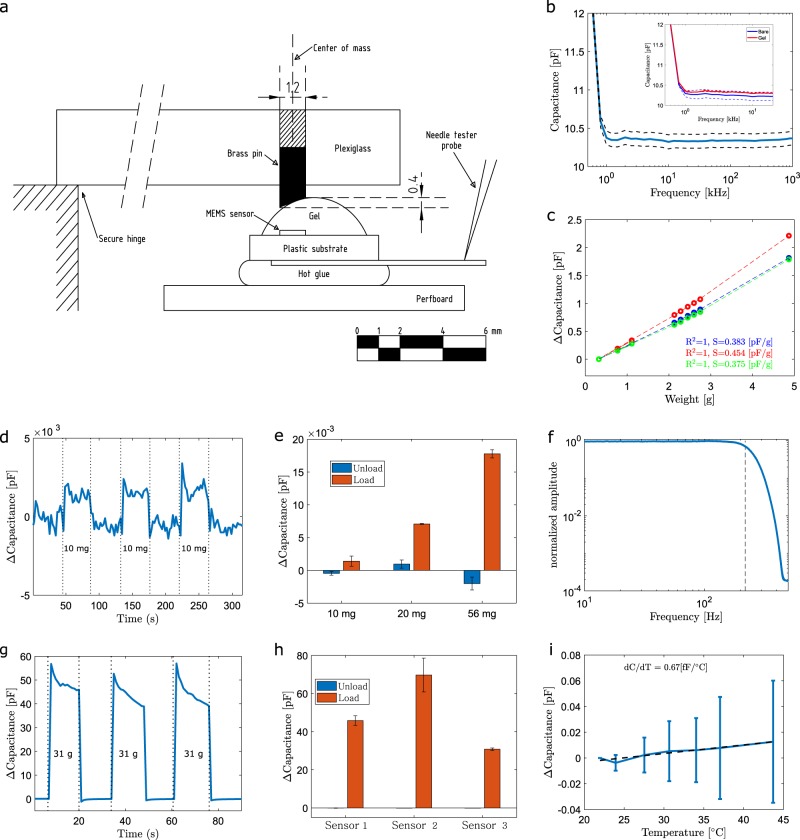
Sensor characterization. **a** Characterization setup. **b** Capacitive properties of modified sensor over frequency. Dashed lines are the standard deviation (*n* = 3). Inset compares pristine and modified sensors. **c** Sensitivity with different weights placed on the top of the sensor (*n* = 3). **d** Time trace of the sensor with sequential loading and unloading of 10 mg weight and **e** repeatability of three sequential loading/unloading of different weights (*n* = 3). **f** Frequency response. **g** Time trace of the sensor when sequentially loaded three times with a large weight mimicking a damaging situation (*n* = 3). **h** Repeatability of three sequential loading/unloading on three sensors and **i** temperature dependence (*n* = 3). All error bars present standard deviation

The sensor element characterization setup is illustrated in Fig. 2a. The enclosed sensor element with two protruding contact pads were attached to a piece of epoxy laminate board. This allowed the assembly to be securely attached to the probe station (Rucker & Kolls 666) bottom plate using a vacuum pump. Needle tester probes were used for connecting the sensor to the LCR meter (Hewlett Packard 4284A). A lift-able piece of plexiglass with a beveled brass tip of 1.2 mm in diameter was used for focusing the weight on the sensor element. Precise amounts of force were applied to the sensor by simply placing a set of different weights on top of the plexiglass while measuring the sensor capacitance with an LCR meter (HP4284A).

First, the capacitive properties of the modified sensor over a broad range of frequency was examined and compared with a pristine element as shown in Fig. 2b. The solid lines present an average of three measurements and dashed lines show the standard deviation. The batch-dependent variation of pristine sensors is clearly larger than the negligible difference measured before and after the modification (see Fig. 2b inset). It can also be observed that the sensor provides a constant capacitance value of 10.4 pF in the frequency range around 1 kHz–1 MHz in atmospheric pressure. The modified sensor characteristics were further evaluated against a simulation model of the pristine sensing element^29,30^ (see Supplementary Information: sensor element capacitance model). The model showed that the capacitance of the modified elements matches that of the predicted value by the model and that there is no reduction in the sensitivity due to gel modification.

In Fig. 2c the response of the sensor was tested by placing several weights on top of the modified element. The sensor’s capacitance follows a parabolic curve in terms of weight. The response curve was fitted with a second-order polynomial resulting in a perfect *R*-squared value indicating good pressure reproducibility between different weights and that the modification does not weaken the element’s properties. An average sensitivity of 0.404 pF/g was obtained when using weights in the range 2–3 g and assuming a linear response.

The resolution of the modified sensor was investigated by placing a minuscule weight on the sensor. As small as 10 mg weight creates a clearly detectable signal as shown in Fig. 2d. This is further illustrated in Fig. 2e, where the averaged values of loading and unloading with different small weights are shown. For each weight (10, 20, 56 mg) three load/unload cycles over three different sensors were averaged. These results show that the differences caused by loading/unloading are easily observable, but also that measurements can be made in absolute terms down to 10 mg.

The frequency response of the modified sensor was examined by subjecting the sensor to an impulse. A fast impulse was created by dropping an elastic ball on top of the sensor that was already under modest static pressure. The frequency response was obtained by a fast Fourier transform (FFT). Before taking FFT the signal was up-sampled to a 1000 Hz. The response is shown in Fig. 2f. The 3 dB point is found around 210 Hz. This provides only a lower limit for the sensor because with this test setup the minimum duration of the impulse is limited. Also, the sampling frequency should be higher to examine the high-frequency response of the element in more detail. However, the results show clearly that the sensor has sufficient bandwidth for the intended application area.

The pristine sensor provides excellent ruggedness and can be exposed to as large as 200 bar pressures without damaging the element.^29^ The ruggedness of the modified elements were studied by repeatedly exposing them to high loads (31 g) causing large, around 50 pF, changes in capacitance as shown in Fig. 2g, h. These tests did not damage the sensors nor did they weaken the sensor characteristics showing that the sensors are rugged and durable.

The temperature dependency of the sensor was studied by heating the sensors to several fixed temperatures between room temperature (22 °C) and 45 °C. A small weight was placed on the sensor mimicking the conditions of the target application. The sensor did not show any significant temperature dependency. From three measured sensors two drifted slightly downward and one upward. The results are shown in Fig. 2i.

### Comparison of non-invasive (NI) and invasive (I) waveforms

The arterial pulse waveform recorded by the MEMS pressure sensors is composed by an initial wave followed by series of reflected waves from the vasculature tree.^31^ We sought to assess the origin and the clinical relevance of the recorded waveform and compared it with the corresponding I catheter pressure recording. In the NI wristband sensing the pressure reading is a sum of three components: (i) atmospheric pressure, (ii) sensor attachment pressure (the pressure that the attachment exerts on the sensor), and (iii) the physiological pressure signal caused by the dilating artery. The atmospheric pressure can be subtracted from the signal with modest ease, but the sensor attachment pressure may vary if the measurement is not controlled rigorously which is unavoidable in most real-life settings. For this reason we concentrate on the shape of the waveforms rather than the absolute amplitude values.

**Fig. 3:**
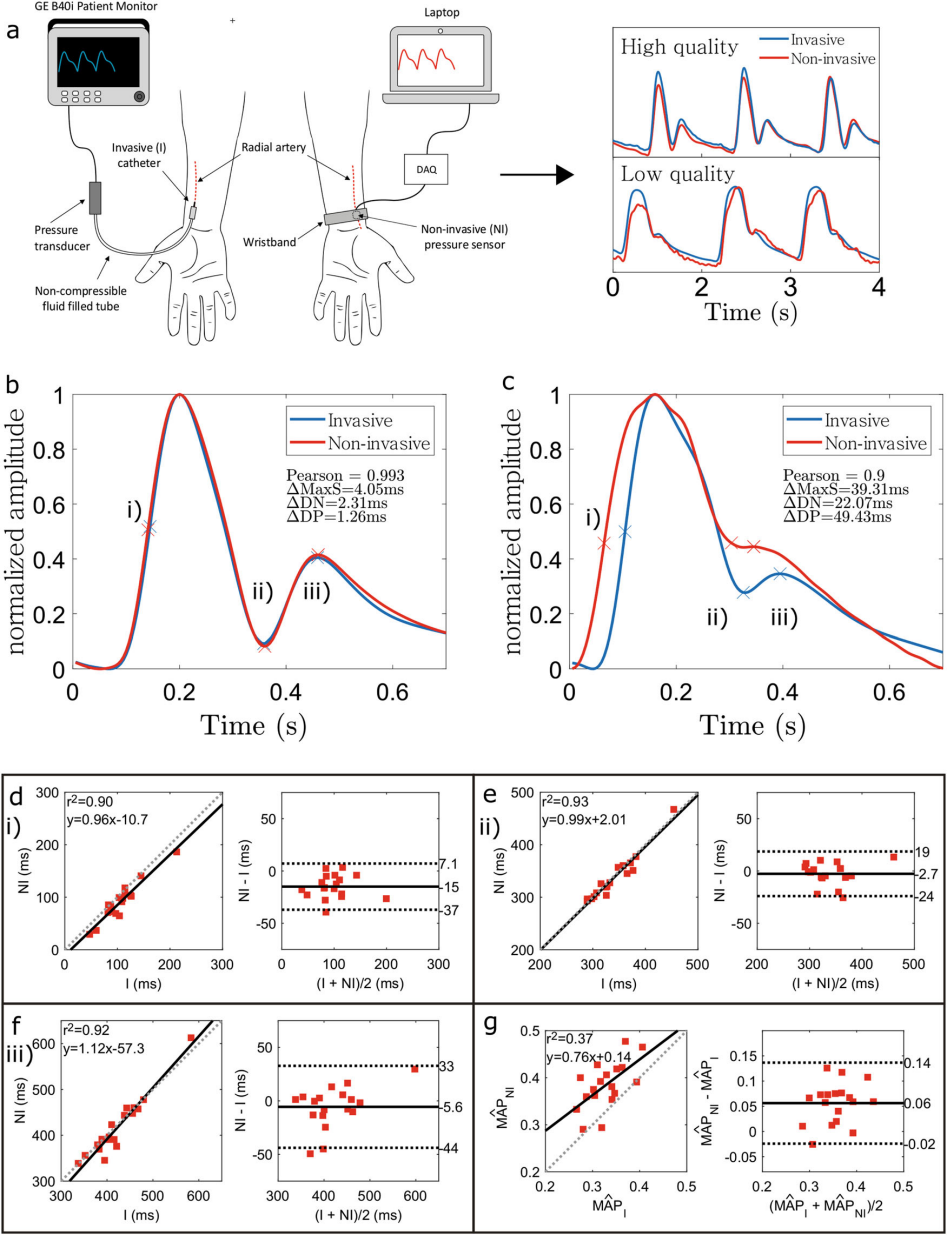
Comparison of non-invasive and invasive waveforms. **a** Measurement setup with the invasive (I) catheter (left) and the non-invasive (NI) wristband (right) along with samples of high-quality and low-quality signals. Comparison of ensemble averaged pulse waveforms of I (blue) and NI (red) pulse waveforms. The waveforms with highest **b** and lowest **c** Pearson correlation coefficient between the I and NI measurements from the study group are shown. **d**–**f** show the correlation and Blandt–Altman plots of the time intervals at (i) maximum slope, (ii) Dicrotic notch, and (iii) diastolic peak, respectively, and **g** compares the normalized MAP values between I and NI measurements. The dashed lines in Bland–Altman plots present the 95% CI

From the ensemble of averaged pulse waveforms we computed the cross-correlation between the NI measurements (DSI) and corresponding I waveforms (DSII) that were aligned from the maximum point. For details on datasets DSI and DSII see the section “Human studies”. Figure 3 illustrates the measurement setup and typical waveforms obtained from I and NI recordings (a), and waveform shape comparison where (b) presents the highest, and (c) the lowest similarity between the I and NI waveforms in the dataset, respectively. The average Pearson correlation over all waveforms in DSII was 0.97 ± 0.02 (mean ± SD) indicating high similarity between all study subjects. Such correlation have not been found with PPG signals,^32^ which might be explained by the fact that PPG does not directly measure pressure waveforms, but mostly arterial blood volume variations.^33^

In addition to the overall waveform similarity, we computed the time differences between the NI and I waveforms in clinically relevant time points.^34,35^ This was examined by computing the time differences between I and NI signals in the following points: (i) maximum slope in the systolic part, (ii) Dicrotic notch, and (iii) diastolic peak. The corresponding linear correlation and Bland–Altman plots are shown in Fig. 3d–f. The *R*^2^ values computed from the linearity plots was above 0.9 for each case. The 95% confidence intervals between the time points from the wearable device and the reference measurements were 22, 21, and 38 ms for (i)–(iii), respectively. From these, the point of maximum slope shows a clear bias towards the NI time point arriving earlier which is a result of the NI pulse waveforms being slightly wider in several measurements. The average fractional errors were roughly 21%, 6%, and 9%. Overall, these results indicate high agreement between the waveforms, but the point of maximum slope has a clear deviation from the reference. This is most likely due to different time constants between the measurement devices that are device construction and post-processing dependent.

In addition to time interval comparison between the waveforms, the waveform amplitude carries plentiful of information. Several parameters such as cardiac output, stroke volume, and vascular resistance, and the variations in the pulse pressure are based on amplitude values of the signal.^36,37^ To evaluate the possibility of a self-referenced measurement system that is able to track relative changes in these parameters we compared the normalized mean arterial pressure (MÂP) between NI and I measurements (Fig. 3g). Several NI measurements had clearly higher MÂP and on average the NI had a higher MÂP estimate. This is due to several low-quality signals caused by poor signal coupling to the element. In higher quality signals, however, the estimates are in close agreement. The average fractional error is about 20%. These errors are systematic within a given measurement and thus indicates that useful continuous measurements based on relative change can be made together with additional signal calibration or by possibly using emerging machine learning-based analysis.^38,39^

Using the developed system, the monitoring of continuous central blood pressure waveform might be possible. Currently, the standard NI method for acquiring aortic waveform is radial tonometry.^40,41^ It requires a trained medical professional to record a short radial waveform sample. The systolic and diastolic pressures measured from brachial artery using a standard blood pressure cuff are matched with the recorded radial pulse. Using specialized signal processing, an estimate of aortic blood pressure waveform is generated. Using our approach, aortic pulse waveform could be created from the radial waveform, e.g. by using a generalized transfer function (GTF).^42^

### Detection of atrial fibrillation

**Fig. 4:**
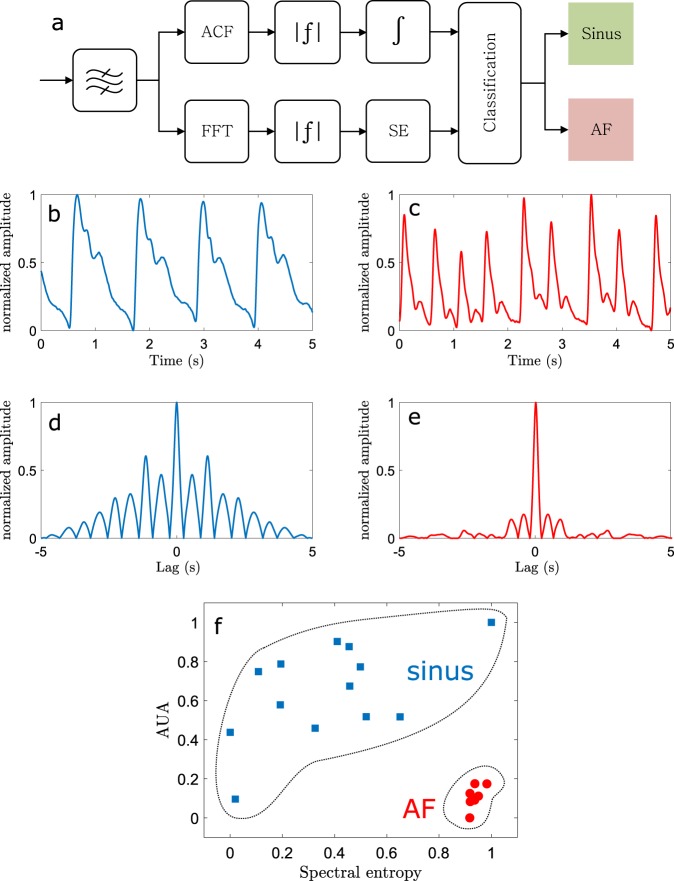
Detection of atrial fibrillation. **a** Pipeline for the atrial fibrillation detection algorithm (band-pass filter, top: autocorrelation, absolute value, integration; bottom: fast Fourier transform, absolute value, spectral entropy; classification). **b** Five second measurement of a healthy 34-year-old male. **c** Typical atrial fibrillation recording of 5 s. **d** and **e** The corresponding absolute value of the autocorrelation of **b** and **c**. **f** Clustering of healthy (*n* = 13) and atrial fibrillation patients (*n* = 7) using time–frequency analysis

Atrial fibrillation is a condition that makes the heart beat irregularly and also leads to large beat-to-beat blood pressure variability.^43^ Both of these characteristics can be seen in Fig. 4 where typical pressure sensor signals of AF patient and a healthy subject are shown. We demonstrate the ability to discriminate between sinus rhythm (DSI) and (persistent) AF patients (*n* = 7) using a time–frequency analysis.^44^ While for the screening of AF, the patients having paroxysmal AF would be more correct test group, it is also acknowledged that separating patients having persistent AF from normal is more difficult because they often have medication that mitigates the differences to the normal rhythm. The pipeline of the automated algorithm is shown in Fig. 4a. The classification algorithm relies on *k*-means clustering with two features: area under autocorrelation (AUA) and spectral entropy. The algorithm is detailed in the section “Algorithm”. The typical time traces of a healthy and AF patient are shown in Fig. 4b, c. Both signals provide clearly distinguishable features and the AF shows, as expected, more irregularity in the heart beats. This is also evident in Fig. 4d, e showing the corresponding absolute value of autocorrelations. The AF signal does not have clear prominent peaks outside zero lag indicating that the rhythm is irregular. Finally, in Fig. 4f, the results from *k*-means clustering is shown. All subjects here are correctly assigned a correct cluster. It is notable that the AF patients are all tightly clustered whereas the healthy subjects are distributed more widely. In both features, at least one healthy subject has a value similar to the AF patients indicating the need for several features for reliable discrimination. As expected, autocorrelation-based values are lower and spectral entropy values are higher on average than the respective values with healthy subjects.

### Heart rate monitoring

**Fig. 5:**
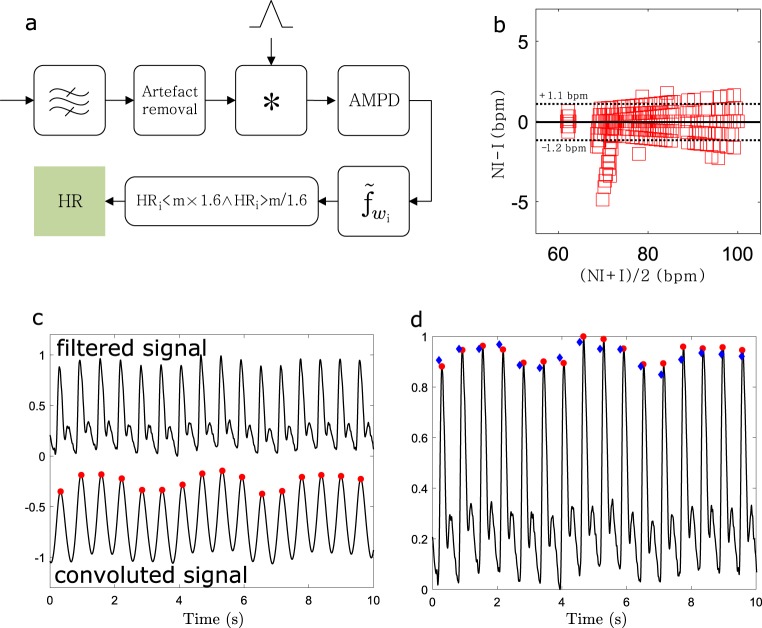
Heartrate detection. **a** Pipeline of the heart rate detection algorithm (bandpass filter, artifact removal, convolution with triangular wavelet, multiscale-based peak detection, median beat interval, accepted HR interval). **b** Bland–Altman plot showing the agreement of heart rates obtained with the non-invasive wearable wristband (NI) and the invasive catheter (I). The dashed lines with corresponding values present the 95% CI. **c** Example of NI signal after band-pass filtering (top) and after convolution with triangle-shaped template (bottom). The red circles present the automatically detected peaks. **d** The found peaks referred back to the band-pass-filtered signal. Red circles and blue diamonds present the peaks from the NI and I (reference) signals, respectively

We considered two independent datasets for assessing the heart rate monitoring capability. These included a group of healthy subjects (DSI) and a group of coronary artery disease patients (DSII). The heart rate algorithm pipeline is shown in Fig. 5a. The algorithm is detailed in the section “Algorithm”. It can extract the beats from DSI with extremely high accuracy due to excellent signal quality. The averaged sensitivity (TRP) and precision (PPV) over all the measurements were 99.1% and 100%, respectively,^45^ without removing any motion artifacts. The beat detection performance of each subject is detailed in Table 1.

**Table 1:** Performance metrics of the beat-to-beat detection (DSI)

ID	HR (bpm)	TPR (%)	PPV (%)
1	90.3	96.9	99.5
2	72.0	100	100
3	67.5	100	100
4	91.8	92.7	100
5	57.5	99.2	100
6	93.4	100	100
7	61.6	100	100
8	107.6	100	100
9	96.9	100	100
10	69.9	100	100
11	60.8	100	100
12	60.2	99.2	100
13	74.3	100	100
Average	77.2	99.1	100

The patient group DSII consisted of patients recovering from a surgery and the results are clearly inferior compared to the results with DSI. One reason is that these patients often had swollen hands making the measurement more difficult. The obtained signal coverage after artifact removal was on average 48 ± 25% (mean ± SD). This clear reduction in coverage compared to DSI was due to artifacts. Visually these artifacts can be divided into a flat signal or high amplitude noise signals. Flat signals are a result of poor signal coupling due to loose attachment and/or a swollen hand, whereas high amplitude noise are due to restless patients. With calm patients there are no artifacts present and signal quality is high, which was indeed observed in DSI and DSIII that did not require any artifact removal. With DSII the artifacts were removed automatically and correctness verified visually which is subject to some interpretation. However, large part of the artifacts are easy to interpret, since the signal is either completely lost or it exhibits high amplitude noise from which the arterial pulse cannot be seen.

Regardless of the artifacts, the averaged 30 s heart rate was accurately obtained as shown in Fig. 5b. The 95% confidence interval indicated with the dashed line in the Bland–Altman plot was (−1.2 to 1.1 bpm) with a mean value of 0.05 bpm. Fig. 5c, d show high-quality signal segment after band-pass filtering and after convolving the signal with triangle-shaped template for easier peak detection.^46^ Found peaks are indicated in the figures with read circles (NI) and corresponding reference peaks with blue diamonds (I).

## Discussion

We developed a wearable real-time monitoring device for continuous arterial pulse recordings for cardiovascular healthcare monitoring. Miniature MEMS pressure sensing elements of size 1.2 mm × 1.2 mm^29^ were covered with a soft silicone gel that effectively mediates the pressure wave from the dilating artery to the sensing element. The hemisphere-shaped gel provides an effective way to obtain high-quality arterial waveforms that are nearly identical in shape when compared to I catheter pressure sensor readings. The sensing element itself is a passive capacitive element and has an extremely low power consumption. The power consumption is mostly dictated by the capacitive currents during the charge–discharge readout. A simple capacitive loss model revealed that the power consumption is only 5 μW when using a 3.3 V power supply and a 100 kHz readout frequency (model details in Supplementary Information: Capacitance measurement principle). This shows that monitoring for long periods with minimal battery sizes is feasible and that the system could be extended to have wireless operation using, e.g. low-energy Bluetooth. The sensors are embedded into a flexible and wearable wristband configuration providing a good sensor to skin contact with a fair user comfort.

It was demonstrated that the device produces a very high-quality signal with healthy subjects as indicated by a near perfect ability to detect heartbeats in continuous monitoring with healthy subjects (DSI). In contrast, the success of the beat-to-beat detection with coronary artery disease patients (DSII) was modest although a high accuracy average heart rate detection was successful after artifact removal. These patients were covering from surgical operation and many had swollen hands limiting the sensors coupling to the artery. It is expected that similar issue prevails with severely obese patients as well. However, from the same challenging dataset (DSII), arterial waveforms that correspond very closely to the I catheter measurements could be extracted along with clinically relevant timing points. Additionally, the capability to detect arrhythmias was tested by classifying sinus rhythm and atrial fibrillation patients. A clustering analysis showed high promise in AF detection. However, the study groups were fairly small and therefore a larger study group is needed to investigate the potential of wearable MEMS pressure sensing for AF detection and to infer the performance compared to ECG, photoplethysmography and mechanocardiography-based methods.^47,48^ We envision that using the presented approach a new class of pressure sensors could be harnessed into a broad range of home monitoring solutions either as a stand-alone solution or integrated with other sensing modalities in the future.

## Methods

### Human studies

*DSI (Healthy group)*: This dataset consisted of 13 healthy volunteers (one female). The demographic of these subjects were as follows (mean, standard deviation): age (34, ±8 years), height (179, ±5 cm), weight (88, ±22 kg), and BMI (27, ±6.0 kg/m^2^). All measurements were NI and carried out using the developed wristband.

*DSII (I group)*: This dataset consisted of 18 volunteers treated at Heart Center Turku University Hospital, who had heart surgery operation the preceding day. The NI and I measurements were taken simultaneously. The location of the arterial cannula prevented taking the NI measurement from the same wrist as the I wave. The NI measurement was taken from left wrist and I measurement from right wrist. The patients were in supine position during the 10 min recording. Study subjects were instructed to be silent and still during the session. The demographic of these subjects were as follows (mean, standard deviation): height (175, ±9 cm), weight (86, ±14 kg), systolic pressure (120, ±15 mmHg), and diastolic pressure (70, ±13 mmHg).

*DSIII (AF group)*: This dataset consisted of seven atrial fibrillation patients admitted to the Heart Center Turku University Hospital. The demographic of these subjects were as follows (mean, standard deviation): height (179, ±5 cm), weight (87, ±10 kg). All measurements were NI and carried out using the developed wristband.

The measurements (DSII, DSIII) were conducted according to the Declaration of Helsinki guidelines at Heart Center, Turku University Hospital, Finland with the permission of Ethical Committee of the Hospital District of Southwest Finland. Written informed consent was collected from all study participants in DSI, DSII and DSIII. The measurements taken from healthy control individuals (DSI) were captured from voluntary participants at University of Turku.

### Measuring devices and systems

The characterization measurements were carried out using a precision LCR meter (HP4284A) controlled by a Labview program. Connection to the element contact pads was made using Rucker & Kolls needle tester. A brass tip was attached to a hinged plastic sheet in order to focus the pressure directly to the sensor element. By using a tip with a beveled head, the effective area of the pressure was consistent between weights and the gel surface of the sensor was not damaged by sharp edges. A vacuum pump was used to make sure that the sensor does not move or tilt during the measurement. The setup illustration is shown in Fig. 2a.

The human subject measurements (DSI and DSIII) and the frequency-response measurements were done with an in-house design. The design employs a capacitance-to-digital converter (PCap01) controlled via Silicon labs EFM32 2-bit ARM Cortex-M3 core- based microcontroller. A Python application was used to acquire the signal from the microcontroller to the PC via the RS232 serial interface. The application was used to display the signal in real-time and save the data for post-processing. Post-processing was carried out with Matlab R14. The sensor board included three pressure sensor elements and the capacitance-to-digital converter was mounted on a printed circuit board (PCB). The board was assembled on an elastic strap and a velcro tape was used to adjust the tightness of it. The sensor board was connected to the microcontroller via flexible copper wires required for powering and for the RS232 interface. Photographs of the readout electronics and accompanying desktop application are shown in Supplementary Information: Measurement devices.

Carescape B40 patient monitor was used for the I catheter measurements (DSII). The data was saved on a file and read using iCollect software and post-processed with Matlab.

### Sensor calibration

The gel-modified sensor elements were calibrated in a pressure chamber using five externally controlled pressure values, *P*_ext_, of 1000, 1100, 1200, 1300, and 1400 mbar. A second-order polynomial fit was created between the *P*_ext_ values and the recorded output signal. This relationship was used to calibrate measured values to atmospheric pressure. This procedure corrects the inherent non-linearity between the measured pressure and capacitance of the elements.

### Algorithm

#### Pre-processing

The signals were filtered with a third-order Butterworth IIR filter with 0.1 and 25 Hz cut-off frequencies that remove bias, trend, and high-frequency noise.

#### Artifact removal

Each signal was segmented into 10 s epochs and for each epoch a single sided FFT is computed. The resulting spectra are smoothed with a moving average filter of 10 samples. Each epoch was integrated using the pre-processing filter pass-band. Epochs that have an integrated value more than 1.3 times the median over all epochs are removed. The median value is updated after each epoch removal yielding always comparable removal threshold. All epochs with a length smaller than 30 s were removed. Artifact removal is used for heart rate monitoring, but not for ensemble averaging described below.

#### Heart rate estimation

The signals after artifact removal are divided into 30 s epochs with 29 s overlap. First, the signal is convoluted with a triangle-shaped wavelet with a width of 0.5 s to simplify the signal shape.^46^ Secondly, the peaks are detected using a multiscale-based peak detection (AMPD) algorithm.^49^ This algorithm constructs a matrix consisting of scale-dependent local maxima. The details for the peak detection are provided in ref. ^45^ Thirdly, the median beat interval is computed over the entire epoch and a corresponding heart rate is computed. Lastly, the heart rates that fall within the range ${\mathrm {HR}}_i\left\langle {1.6 \times m \wedge {\mathrm {HR}}_i} \right\rangle m{\mathrm{/}}1.6$ are included,^46^ where *m* is the median value over one recording.

#### Average waveform

The I and NI signals obtained from two independent measurement devices were synchronized by computing the lag between the two signals via cross-correlation and shifting the signals in relation to others by the lag time of the highest correlation. The peaks in the I measurements were used as fiducial points for waveform ensemble averaging by creating a window around each peak for one full waveform extraction, referred as template. The same fiducial time points were used for NI measurement to ensure that the ensemble averaging is obtained from the corresponding templates in both signals. The templates that had a correlation with the median waveform (obtained from the entire signal) <0.98, were omitted, or 0.9 when all templates were disregarded. This template selection was used independently to both I and NI signals. The remaining templates were ensemble-averaged resulting in the final waveform. For quantitative comparison, a Pearson correlation coefficient was computed between each corresponding template in both signals after which the coefficients were averaged. This method is applied for the full length signals without artifact removal.

#### Waveform analysis

The obtained waveforms (both I and NI) were up-sampled to 1000 Hz and normalized to have amplitude values from 0 to 1. First-order and second-order derivatives were computed from the ensemble-averaged pulse waveform. The maximum amplitude was considered as the systolic peak. The Dicrotic notch was found by evaluating the zero crossing in the first-order derivative signal occurring after the systolic peak. Diastolic peak was the local maxima in the mean waveform occurring after the Dicrotic notch. The normalized MÂP was computed from the normalized pulse waveforms by first computing the area under the curve via integration and by dividing this result with the length of the waveform.

#### Detection of atrial fibrillation

After pre-processing, the signals were divided into 5 s non-overlapping epochs without any artifact removal. A time–frequency analysis was carried out to each epoch. (i) autocorrelation was computed to each epoch and the results were normalized to have a maximum amplitude of 1. Subsequently, a sample-wise ensemble average was computed and the absolute value was taken. Finally, the AUA was computed via integration. (ii) The spectral entropy was computed for each epoch by first taking the |FFT| from each epoch. The spectra were normalized to have an area of one. The spectral entropy is computed from the resulting probability density function $p\left( f \right)$ by ${\mathrm {SE}}_{{\mathrm {epoch}}} = - {\sum} {p\left( f \right)} \times \ln \left( {p\left( f \right)} \right)$. Each epoch has one entropy value assigned to it and the final entropy value SE is average over all SE_epoch_.

These time and frequency features were used as input features in *k*-means clustering. Both features were normalized to have values between 0 and 1 by $\left( {v - \min \left( v \right)} \right)/\left( {\max \left( v \right) - \min \left( v \right)} \right)$, where *v* presents the feature vector. The measurements were clustered using the cosine distance.

### Reporting summary

Further information on research design is available in the Nature Research Reporting Summary linked to this article.

## Supplementary information

Supplementary Information

reporting summary

Data Set 1

Data Set 2

Data Set 3

Data Set 4

Data Set 5

Data Set 6

Data Set 7

Data Set 8

Data Set 9

Data Set 10

Data Set 11

Data Set 12

Data Set 13

## Data Availability

The data concerning healthy subjects (DSI) is openly available and can be downloaded as part of the Supplementary Information. DSII and DSIII that support the findings of this study are available from Turku University Central Hospital (TYKS) under clinical permissions, acquired for the current study, but it is not currently publicly available.

## Supplementary information

**Supplementary information** accompanies the paper on the *npj Digital Medicine* website (10.1038/s41746-019-0117-x).

## Code availability

All computer code supporting the presented findings are detailed in the “Methods” section. All code was written with Matlab R14. The computer code of this study are available from the corresponding author upon reasonable request.
