# Supplementary material for: Clinical assessment of a non-invasive wearable MEMS pressure sensor array for monitoring of arterial pulse waveform, heart rate and detection of atrial fibrillation
Source: NPJ Digit Med. 2019 May 14;2:39. doi: 10.1038/s41746-019-0117-x (PMC6550190; doi:10.1038/s41746-019-0117-x)
Supplement: Supplementary file 1 — Supplementary Information [file 41746_2019_117_MOESM1_ESM.pdf]

Supplementary Information

Clinical Assessment of a Non-Invasive Wearable MEMS Pressure Sensor Array for Monitoring of Arterial Pulse Waveform, Heart rate and Detection of Atrial Fibrillation

Matti Kaisti[1,2], Tuukka Panula[1], Joni Leppänen[3], Risto Punkkinen[1], Mojtaba Jafari Tadi[1], Tuija Vasankari[4], Samuli Jaakkola[4], Tuomas Kiviniemi[4,5], Juhani Airaksinen[4], Pekka Kostiainen[3], Ulf Meriheinä[3], Tero Koivisto[1], Mikko Pänkäälä[1]*

Measurement devices

A screen capture of the graphical user interface used to monitor and save signals from the three sensors in the array simultaneously is shown in Figure S1. Figure S2 is a photograph of the device used to connect the wristband device to PC.

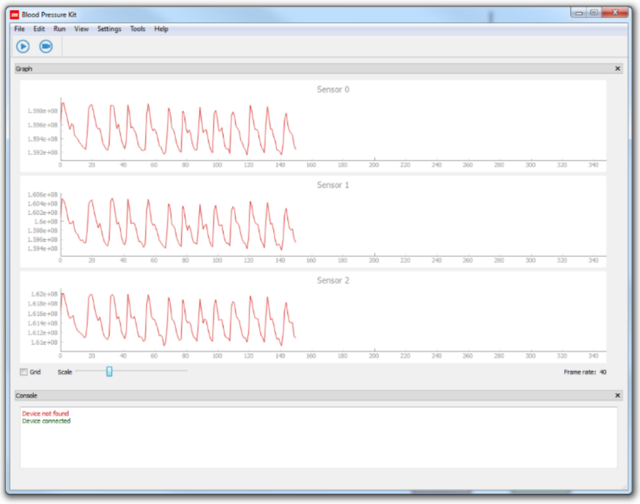

Figure S1. Graphical User Interface (GUI) for the non-invasive sensor. The user can see the downsampled waveforms of each sensor.

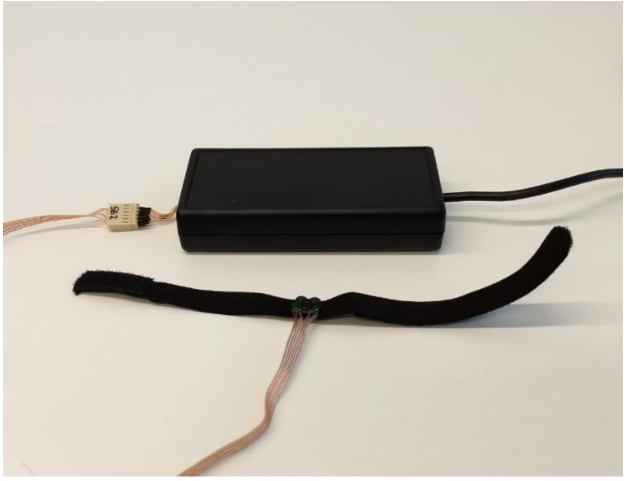

Figure S2. Non-invasive sensor wristband and the encased interface electronics (Silicon labs EFM32 microcontroller board).

Sensor element capacitance model

The bending of the diaphragm towards the stationary electrode happens in a certain manner that can be expressed via a multiple parallel plate model[1]

$$C(p) = C_{00} + \frac{C_0}{1 - \frac{C_0}{K}p} + \frac{aC_0}{1 - \frac{C_0}{bK}p}$$

where C00 is a stray capacitance, C0 is proportional to the inverse of the distance between the capacitor electrodes. The parameters a and b are constants and define the properties of a specific element. C00, C0 and K are statistically independent and vary due to the manufacturing process within a specified range. The values required for explaining the sensors capacitive properties are given in TableS1. Results from a Monte Carlo simulation using the tolerance values (uniform distribution) were compared to atmospheric pressure measurements of the fabricated tactile sensors as shown in Figure S3 (left). Simulation was repeated 10 000 times. In the figure the min and max capacitance values from the model are shown using a pressure range from 100 kPa to 140 kPa. The same range was used for the atmospheric measurements where the change in pressure was created in a chamber and measured with a manometer. As shown, the measured sensors had lower offset value than the model average, but clearly within the tolerance of pristine sensors. To further evaluate the possible change in the sensitivity, each response was shifted to start from zero capacitance, shown in Figure S3 (right). It is clearly seen that the measured sensitivity is around the expected sensitivity, further proving that the modification of the elements does not weaken its sensing properties.

Table S1. Capacitance model parameter.

Parameter	Nominal value	Tolerance
a	1.759	
b	1.485	
C_{00}	0.9 pF	±0.22 pF
C_0	2.24 pF	±0.13 pF
K	509	-103…-112

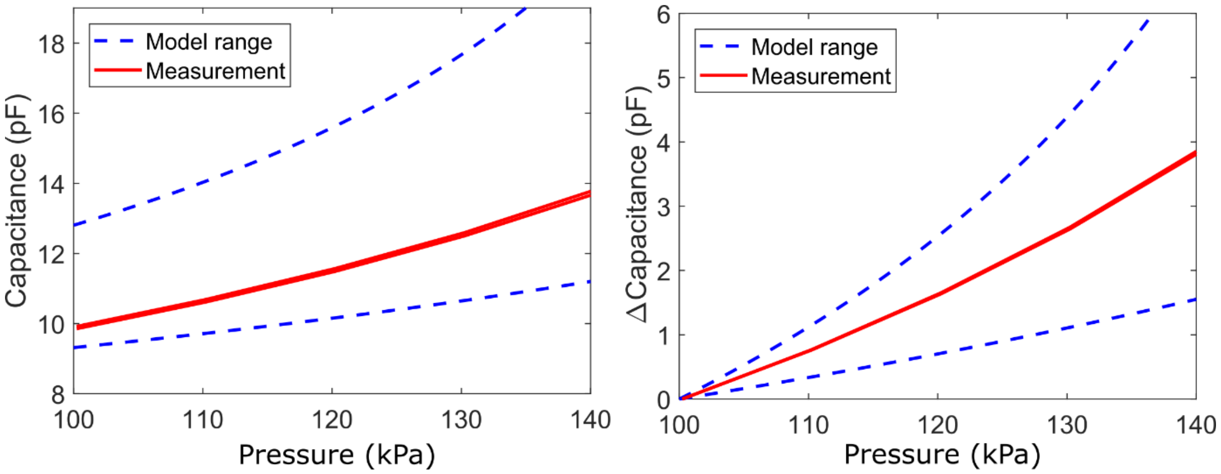

Figure S3. Left: Pristine element capacitance value simulation showing a minimum and a maximum from a Monte Carlo simulation. Right: Pristine element capacitance value simulation showing a minimum and a maximum from a Monte Carlo simulation with each measured response shifted to start from zero capacitance.

Capacitance measurement principle

A single AMS PCap04 capacitance-to-digital converter is used to measure the three sensor capacitances. The measurement is based on measuring the discharge time on RC-networks. Each sensor capacitance is measured in its dedicated cycle. First the capacitor is loaded to full charge. Then PCap04 uses user configurable discharge resistors to discharge the capacitor. The actual measurement happens from the start of the discharge until a threshold voltage is reached. Finally the capacitor is grounded.

An analysis on power consumption of the element can be made by considering the capacitive switching losses. The element itself is passive with very low leakage currents and majority of power consumed is due to charging and discharging of the capacitive element with high frequency. A simple capacitive switching loss model is described with

$$P = \frac{C \times V^2 \times f}{2}$$

where C is the capacitance of the element, V is the full charge/discharge voltage and f is the charging frequency. The models reveals as shown in Figure S4 that with typical supply voltage of 3.3 V and 100 kHz read-out frequency the consumption is a modest 5 µW and can be even further reduced by lowering the frequency and supply voltage. This analysis is also conservative as it assumes full charge/discharge on each cycle.

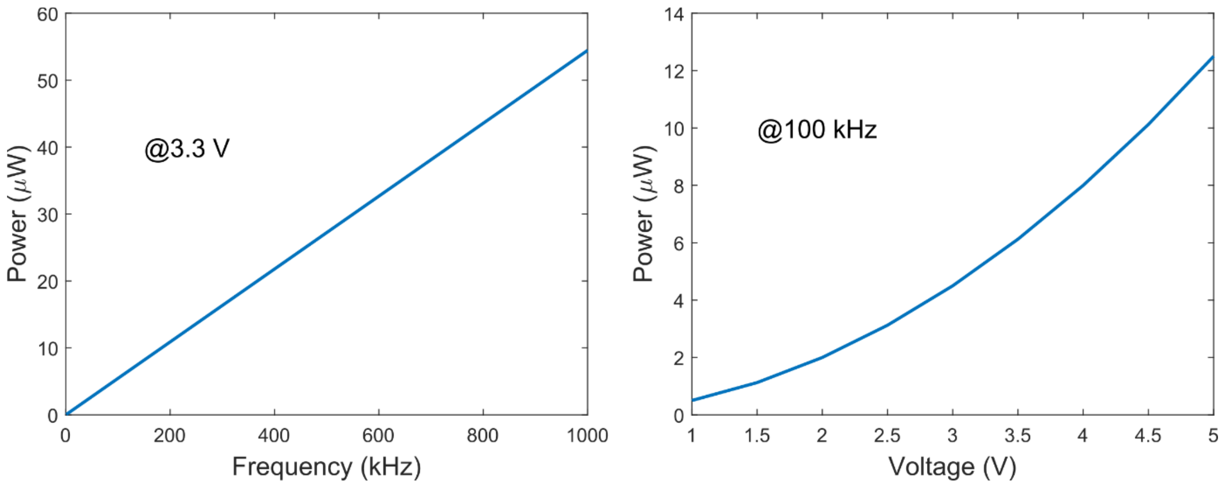

Figure S4. Left: Simulated capacitive losses during sensor read-out as a function of frequency. Right: Simulated capacitive losses during sensor read-out as a function of supply voltage.

References

1. *Product family spesification, SCBH10 Series Pressure Elements,Doc. No. 82 1250 00 B.* (2017).
